# Gait-modifying effects of augmented-reality cueing in people with Parkinson’s disease

**DOI:** 10.3389/fneur.2024.1379243

**Published:** 2024-04-09

**Authors:** Eva M. Hoogendoorn, Daphne J. Geerse, Annejet T. van Dam, John F. Stins, Melvyn Roerdink

**Affiliations:** Department of Human Movement Sciences, Faculty of Behavioural and Movement Sciences, Vrije Universiteit Amsterdam, Amsterdam Movement Sciences, Amsterdam, Netherlands

**Keywords:** Parkinson’s disease, Augmented Reality, Mixed Reality, gait parameters, visual cueing, HoloLens 2, Magic Leap 2

## Abstract

**Introduction:**

External cueing can improve gait in people with Parkinson’s disease (PD), but there is a need for wearable, personalized and flexible cueing techniques that can exploit the power of action-relevant visual cues. Augmented Reality (AR) involving headsets or glasses represents a promising technology in those regards. This study examines the gait-modifying effects of real-world and AR cueing in people with PD.

**Methods:**

21 people with PD performed walking tasks augmented with either real-world or AR cues, imposing changes in gait speed, step length, crossing step length, and step height. Two different AR headsets, differing in AR field of view (AR-FOV) size, were used to evaluate potential AR-FOV-size effects on the gait-modifying effects of AR cues as well as on the head orientation required for interacting with them.

**Results:**

Participants modified their gait speed, step length, and crossing step length significantly to changes in both real-world and AR cues, with step lengths also being statistically equivalent to those imposed. Due to technical issues, step-height modulation could not be analyzed. AR-FOV size had no significant effect on gait modifications, although small differences in head orientation were observed when interacting with nearby objects between AR headsets.

**Conclusion:**

People with PD can modify their gait to AR cues as effectively as to real-world cues with state-of-the-art AR headsets, for which AR-FOV size is no longer a limiting factor. Future studies are warranted to explore the merit of a library of cue modalities and individually-tailored AR cueing for facilitating gait in real-world environments.

## Introduction

External cueing is a well-established compensation strategy (1, 2) for improving gait (e.g., step length and gait speed) and ameliorating freezing of gait (FoG) in people with Parkinson’s disease (PD) (3–5). Although the precise underlying neural mechanisms of cueing remain unclear, there is consensus on the notion that locomotor control is shifted from automatized (without cues) toward goal-directed (with cues) control (2, 4). External cues, defined as spatial or temporal stimuli (3), are typically classified as either visual, auditory, or somatosensory. Visual cues that have been employed include spatial, transversal lines taped on the ground as a target for foot placement, but can also be implemented with a body-worn laser light projecting on the floor (6–8). Auditory (e.g., metronome) (9, 10) or somatosensory (e.g., vibrating wearable devices) (11–13) cues provide a temporal rhythm for step synchronization.

Even though positive findings have been consistently reported, existing cueing modalities all face practical challenges; physical, visual cues are location-bound while body-worn laser lights are less visible in bright environments. Also, auditory cues can interfere with relevant environmental sounds, and somatosensory devices may not be suitable for people with sensory impairments (12), to name a few. Also, the coupling strength between steps and cues varies with cueing modalities: the stronger gait is tied to the cue, the greater the gait-modifying effects of the cues, yielding superior effects for visual cues (14, 15), followed by auditory and somatosensory cues. As the response of people with PD to cueing is highly variable [e.g., a person with PD showing responsiveness to 3D cues, but not to 2D cues (16)], flexibility is required for tailoring cues to this heterogeneity, as well as to individual-specific gait characteristics (1, 17), which may be challenging for some existing one-size-fits-all types or forms of cueing.

There is thus a clear need to enhance cueing in terms of its modality (focus on visual cues for its superior coupling, perhaps even multimodal to benefit from combined spatiotemporal cues), delivery (wearable to make cues available anywhere, anytime), flexibility (select type of cue that works best for a given person or situation), and personalization (adjusted to individuals’ gait characteristics and needs). One emerging technology to accommodate these requirements may be Augmented Reality (AR) (18, 19), involving software applications for wearable headsets or glasses through which the user’s environment can be augmented with visual holographic, digital objects. Recent studies have shown potential for providing AR cueing and training programs for people with PD to improve gait and balance (20–23), which raised interest for implementing cues in AR. AR breaks the boundaries of physical visual cues with the possibility of projecting holographic visual cues anywhere, anytime. Moreover, the digital nature of the cues implies that they can be easily adapted in various respects (e.g., length, height, depth, color, motion), allowing for cue flexibility and personalization. Even though early AR cueing research in people with PD with the first-generation AR headsets did not find any significant improvements on FoG, the results were still encouraging as subjective benefits of AR cueing are often reported (23–25). The lack of positive findings may be related to the limited AR field of view (AR-FOV) of the AR headsets (18, 23, 26, 27), an insufficient familiarization period to AR headsets (23), the fact that only one specific visual cue was implemented (1, 17), or the emphasis on FoG as an outcome measure instead of other valuable gait characteristics like gait speed and step length, susceptible to improvement with AR cueing (20, 25, 28, 29). In the present study we address these issues.

The aim of this study is to evaluate the gait-modifying effects of AR cueing in people with PD. We implemented several types of cues like speed lines, stepping stones, and 3D hurdles, varied their speeds, inter-cue distances, and heights, and quantified whether this led to adjustments in gait speeds, step lengths, and step heights. The primary objective of this study was twofold: (i) to examine if people with PD were able to modify their gait to AR and real-world cue variations and (ii) whether the adjustments were equivalent to what was imposed with the cues. We also explored additional benefits offered by AR, like sound augmentation of visually cued steps to improve their action relevance (30) and applying visual cues in the air at eye height to prevent a downward head orientation, thereby promoting an upright posture. A secondary objective was to examine the effect of the different AR-FOV sizes of two state-of-the-art AR headsets [i.e., Microsoft HoloLens 2 (HL2) has a smaller vertical AR-FOV than Magic Leap 2 (ML2)] on the gait-modifying effects of AR cues and the required head orientation to interact with them.

## Materials and methods

### Participants

This study was approved by the accredited Medical research Ethics Committees United (MEC-U), the Netherlands (R22.076, NL82441.100.22). Individuals with PD who participated in a clinical feasibility study on home-based gait-and-balance exergaming with AR headsets (21, 31) were invited to participate in the current study. The benefit of recruiting participants from this clinical feasibility study was that participants were already familiar with AR headsets for at least 3 weeks. Exclusion criteria were additional neurological diseases and/or orthopedic problems seriously interfering with gait-and-balance function, insufficient physical capacity or cognitive and/or communicative inability to understand instructions and participate in the tests, visual or hearing impairments (after corrective aids), severe visual hallucinations or illusions, inability to walk independently for 30 min, and no stable dosages of dopaminergic medication (21). All participants signed written informed consent before participation. Two AR headsets, HL2 and ML2, were block-randomized over participants.

### Experimental set-up and procedure

The experiment was performed on the Interactive Walkway, a 10-meter walkway instrumented with an integrated multi-Kinect v2 set-up for markerless registration of 3D full-body kinematics during walking. The Interactive Walkway has been validated for deriving gait parameters of people with PD (32) and was recently also used for validating the HoloLens 1 for quantifying spatiotemporal gait parameters (33). External real-world visual cues can be projected onto the 10-meter walkway, such as projected speed-line cues and 2D stepping targets (34). The holographic AR cues were presented in state-of-the-art optical see-through AR headsets, HL2 and ML2, of which ML2 has a substantially larger vertical AR-FOV [horizontal × vertical: 45° × 55° (35)] than HL2 [43° × 29° (36)], using a purpose-specific software application developed by Strolll Limited. Both AR headsets recorded headset positions and orientations in 3D, with higher orientation values representing more downward headset orientations.

First, participants walked twice on the Interactive Walkway without the AR headset at self-selected comfortable walking speed to determine their preferred gait characteristics (i.e., gait speed and step length). Next, participants walked the Interactive Walkway again while wearing the AR headset (without AR cues) to determine if wearing it influenced participants’ gait parameters, which was not the case: gait speed and step length were both statistically equivalent for walking with and without the AR headset, allowing for a fair comparison between AR and real-world cueing conditions while using the AR headset to register 3D head positions and orientations.

**Figure 1:**
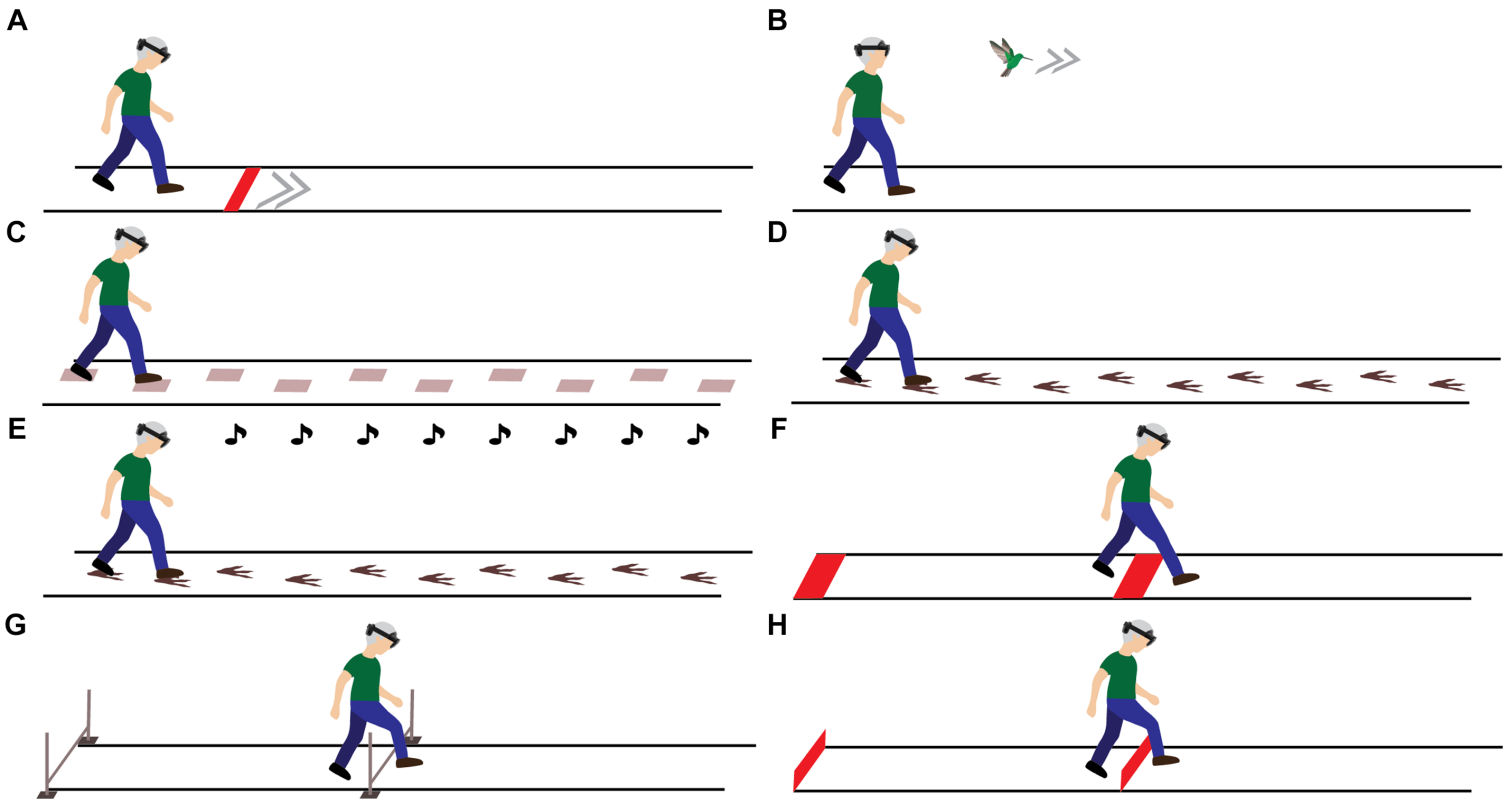
Visualization of the experimental set-up and tasks to modify gait speed [real-world and AR speed line **(A)** and AR speed bird **(B)**], step length [real-world stepping targets **(C)** and AR dinosaur footprints serving as stepping targets **(D)** with acoustic step augmentation through mud sounds **(E)**], crossing step length [real-world and AR 2D obstacles **(F)**] and step height [physical 3D hurdles **(G)** and AR 3D hurdles **(H)**].

**Figure 2:**
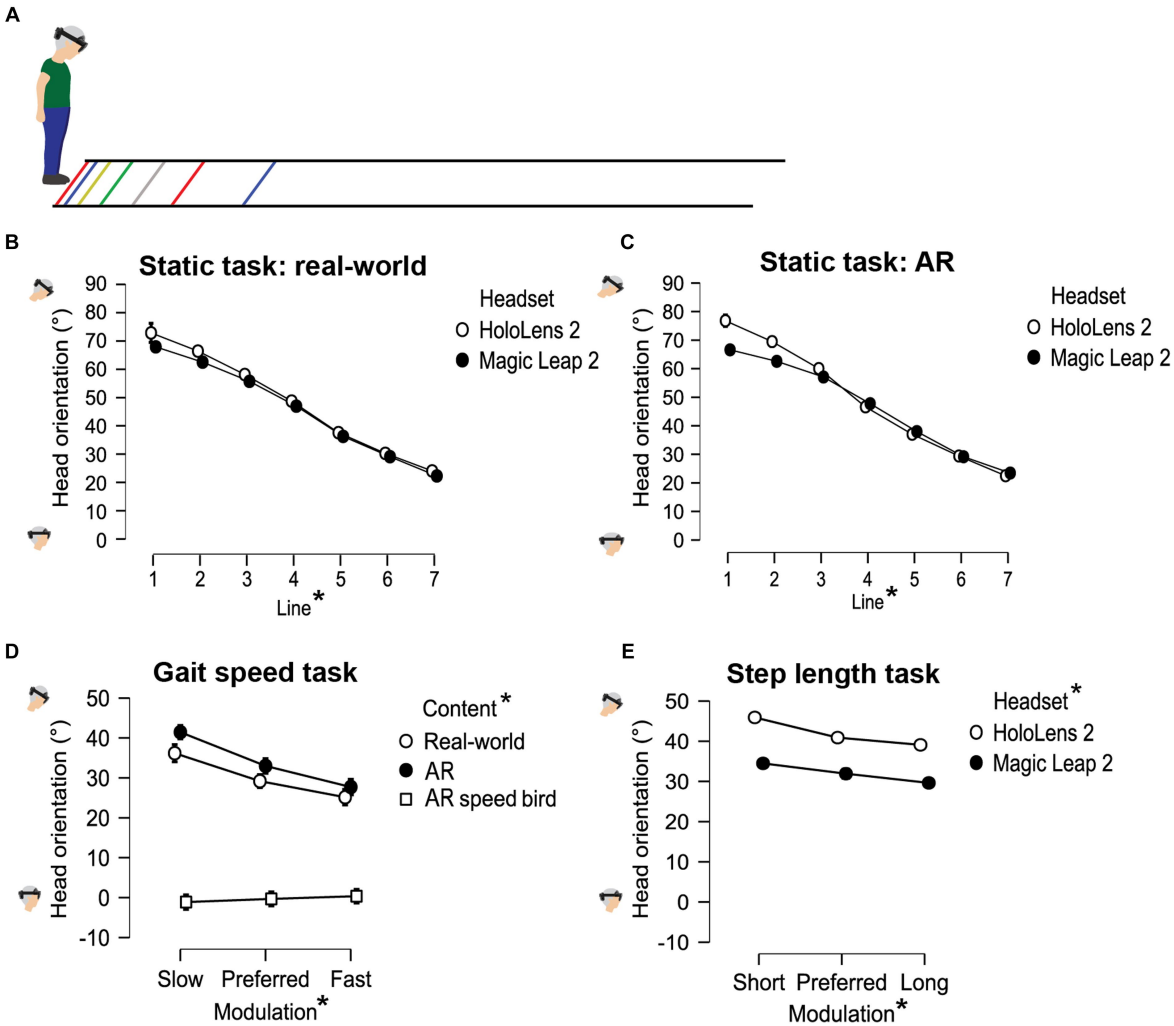
Visual representation of the head orientation during the static task **(A–C)** and during gait-speed **(D)** and step-length **(E)** modulations. * denotes a significant main effect between conditions (*p* < 0.05).

Subsequently, a static task (quiet standing) and several walking tasks (Figure 1) were all performed once. Conditions of the walking tasks were randomized over participants: (1) content (real-world or AR) and (2) modulation (slow/short/small/low or preferred/medium or fast/long/large/high). Tasks were performed in a fixed order, resulting in two static trials and 30 walking trials for each participant to modulate (see Supplementary Video):

1. *Head orientation*: Participants were looking from a stand-still position for 5 s at several projected lines located on the ground at specific distances (10, 30, 60, 100, 150, 210, and 280 cm; Figure 2A) to determine the head orientation required for looking at either real-world cues or AR cues.
2. *Gait speed*: Participants were instructed to walk behind a real-world and AR red speed line visible on the ground along the walkway (i.e., speed cue), moving at different speeds relative to the participant’s baseline speed [i.e., baseline-20 cm/s (slow), baseline (preferred), and baseline + 20 cm/s (fast); Figure 1A]. Consecutively, an AR flying bird at eye height was implemented as an alternative for commonly used floor-based visual speed cues (Figure 1B).
3. *Step length*: Participants stepped onto real-world 2D stepping targets (Figure 1C) or AR dinosaur footprints (Figure 1D), both at varying inter-cue distances [baseline-15 cm (short), baseline (preferred), and baseline+15 cm (long)]. Consecutively, mud sounds were played to AR dinosaur footprints when stepping onto them (step augmentation; Figure 1E).
4. *Crossing step length*: Participants were instructed to step over real-world and AR 2D obstacles, located at the start and halfway of the walkway, which varied in depth [15 cm (small), 30 cm (medium), and 45 cm (large) deep; Figure 1F].
5. *Crossing height*: Participants were instructed to cross real-world and AR 3D hurdles, located at the start and halfway of the walkway, that varied in height [5 cm (low), 10 cm (medium), and 15 cm (high); Figures 1G,H].

### Data and statistical analysis

For the walking trials, pre-processing of Interactive Walkway full-body kinematic data followed established procedures (37, 38) using Matlab R2023a (39). The stepping-height trials were not analyzed since we were not able to accurately record vertical position data due to set-up restrictions. 10 out of the remaining 525 walking trials showed missing data due to communication issues with the Kinect sensors. In 4 of these trials, gait parameters could still be determined over a smaller portion of the walkway, the other 6 trials were excluded. Missing data was excluded analysis-by-analysis. Two participants were excluded from the step-length task because of experiment failure (i.e., inconsistent imposed step lengths across real-world and AR conditions).

For the primary objective, outcome measures that were calculated were gait speed [i.e., distance traveled between 3 and 9 meters on the walkway, to allow for adaptation to the speed cue, divided by the time elapsed using the data of the spine shoulder (37)], step length [i.e., median of the differences in the anterior–posterior direction of consecutive step locations between 1 and 9 m on the walkway (37)], crossing step length at gait initiation (i.e., difference between the first step location in the anterior–posterior direction and the median location of the leading limb before the start of the trial) and crossing step length halfway along the walkway (i.e., difference in the anterior–posterior direction of the first step location after 5 meters and the last step location before 5 meters). To determine whether participants adjusted their gait to the cues, gait speed and step length were both subjected to a 2 × 3 × 3 [Headset × Content (real-world, AR, AR speed bird/AR with sound) × Modulation (slow/short, preferred, fast/long)] mixed ANOVA. To determine whether participants’ performed gait speeds and step lengths were not different to what was imposed, two one-sided *t*-tests (TOST) were conducted in Jamovi [Jamovi 2.3.28, utilizing the TOSTER module which allows us to establish equivalence (40)]. This allows researchers to provide support for the null hypothesis (i.e., no meaningful effect), within a frequentist framework (40). Limits of the TOST were set at 25% of the imposed modulations (i.e., 5 cm/s for speed and 3.75 cm for step-length modulations) acknowledging some natural gait variability (41). Observations within these limits are considered equivalent (i.e., no meaningful difference), but may still be statistically different (40). Crossing step length was subjected to a 2 × 2 × 2 × 3 [Headset × Content (real-world, AR) × Location (gait initiation, halfway along the walkway) × Modulation (small, medium, large)].

For the secondary objective, the headset orientation during the static trial was determined as the median headset orientation for the duration participants were looking at a specific line, corrected for baseline orientation (defined as the headset orientation when looking straight ahead at the start and end of the trial). The headset orientation was subjected to a 2 × 2 × 7 mixed ANOVA [Headset × Content (real-world or AR) × Distance (line 1 to 7)]. Two participants did not execute the static task because of the inability to maintain a static posture and difficulties with following the instructions. For the walking trials to modulate gait speed and step length, the median headset orientation was calculated between 3 and 7 meters, again after subtraction of the baseline headset orientation. Median headset orientations were subjected to a 2 × 2 × 3 [Headset × Content (real-world or AR) × Modulation].

Except for the TOST, all statistical analyses were performed in JASP (42). For the mixed ANOVAs, the assumption of sphericity was verified according to Girden (43). The Huynh-Feldt correction was applied if Greenhouse–Geisser’s epsilon exceeded 0.750, otherwise the Greenhouse–Geisser correction was used. Effect sizes were quantified with *ƞ*_p_^2^. The main effects and relevant significant interactions for our objectives were further explored with post-hoc *t*-tests using a Bonferroni correction. Possible significant three-way or four-way interactions, that were deemed relevant for the objectives, were further examined with two-way ANOVAs for each factor. All data underlying the statistical analyses are available in Supplementary Table 2.

## Results

### Participants

Twenty-one people with PD were included in the study. There were no significant between-group differences in any of the participant characteristics, including age (mean [range]: 63 ± 8.6 [51–74] and 69 ± 8.3 [53–82] years of age) and Modified Hoehn and Yahr stage (stages 2/2.5: 7/4 and 6/4). See Supplementary Table 1 for an elaborate overview of participant characteristics.

### Can we modify gait with AR cues?

**Figure 3:**
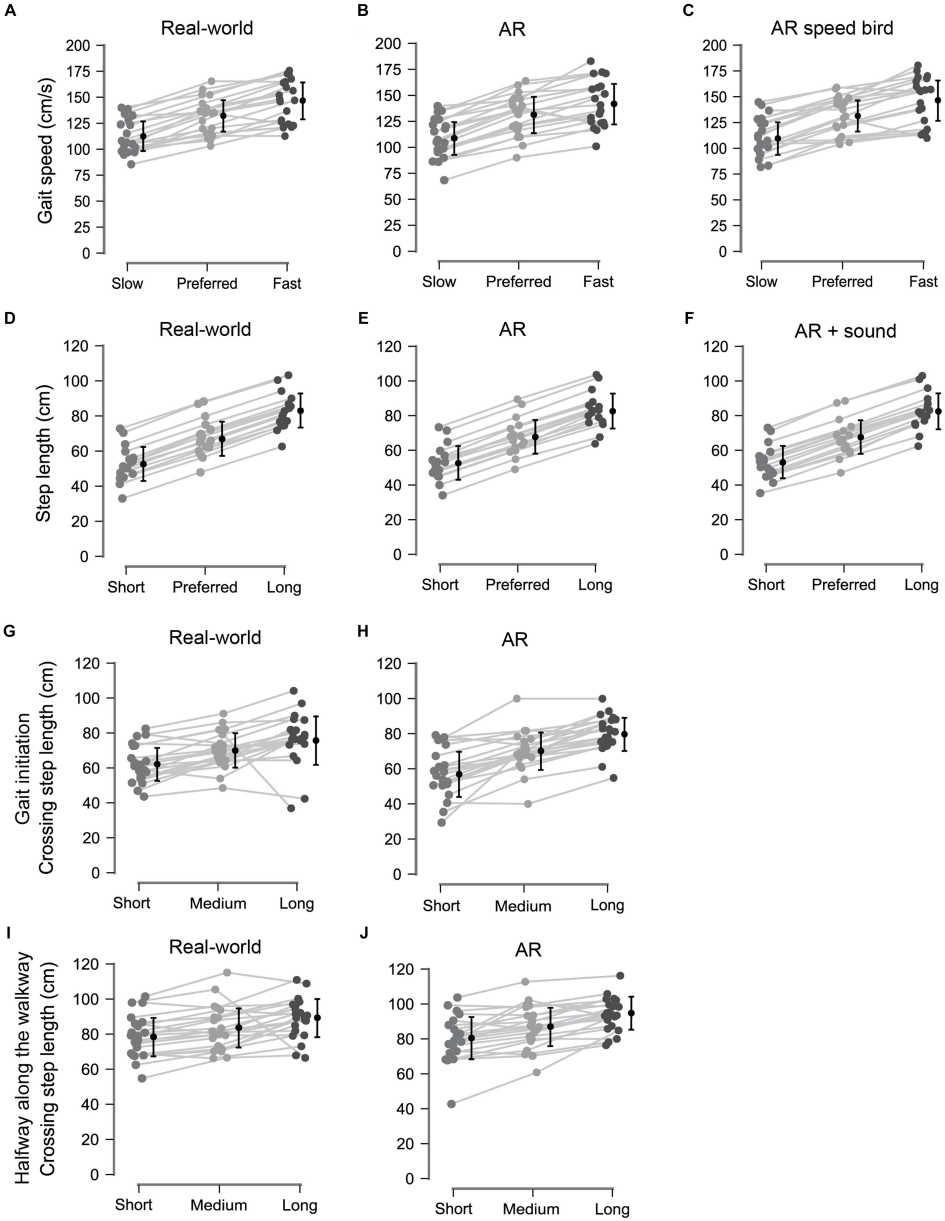
Data visualization of the modulation effects; gait speed **(A–C)**, step length **(D–F)**, and crossing step length at gait initiation **(G,H)** and halfway along the walkway **(I,J)** all differed significantly between all modulation levels.

People with PD modified their gait to the cues with different executed gait speeds or (crossing) step lengths for different modulation levels. Participants increased their gait speed, step length and crossing step length when faster or larger steps were imposed by the cues. This was evidenced by significant main effects of Modulation for gait speed [*F*(1.55,27.86) = 115.509, *p* < 0.001, *ƞ*_p_^2^ = 0.865], step length [*F*(2.00,31.26) = 14285.076, *p* < 0.001, *ƞ*_p_^2^ = 0.999] and crossing step length [*F*(1.29,24.53) = 63.860, *p* < 0.001, *ƞ*_p_^2^ = 0.771], with significant post-hoc differences between all three modulation levels (*p_bonf_* < 0.05; Figure 3).

For crossing step length, larger crossing steps were taken halfway on the walkway (85.35 ± 12.82 cm) than at initiation (69.36 ± 14.14 cm; *p_bonf_* < 0.001), following from a significant interaction with the factor Modulation [i.e., Modulation × Location interaction, *F*(1.41,26.79) = 8.293, *p* = 0.004, *ƞ*_p_^2^ = 0.304], with significant post-hoc differences between the locations of the cue for all three modulation levels (*p_bonf_* < 0.001), accompanied by a main effect of Location [start vs. halfway; *F*(1,19)=143.048, *p* < 0.001, *ƞ*_p_^2^ = 0.883].

For all gait parameters, the modifications did not differ between real-world and AR cues as effects with the factor Content were generally absent, suggesting that gait speed, step length, and crossing step length can be modified with both real-world and AR cues. Exceptions were a significant Content × Headset interaction for the step-length task [*F*(1.85,25.84) = 8.135, *p* = 0.002, *ƞ*_p_^2^ = 0.368] and a significant Content × Location interaction for crossing step-length task [*F*(1,19)=7.773, *p* = 0.012, *ƞ*_p_^2^ = 0.290], although without relevant significant post-hoc comparisons.

### Are gait adjustments equivalent to what was imposed?

The TOST was performed to determine if the observed gait modifications were equivalent to what was imposed by the cues. The performed gait speeds were generally not equivalent to the imposed gait speeds, for both real-world and AR cues alike, except for the condition with the slower-than-preferred AR speed cue (Table 1). Participants seemed to walk slightly faster than imposed with slower imposed speeds. In contrast, step length was statistically equivalent to what was imposed for all conditions.

**Table 1:** TOST statistics of gait-speed and step-length modulations.

		Imposed	Executed	*t*-test			TOST lower			TOST upper			Equivalence*
		Mean ± SD	Mean ± SD	*t*	df	*p*	*t*	df	*p*	*t*	df	*p*	Yes/No
**Gait speed**													
Real-world speed line	Slow	107 ± 16.40	112 ± 16.30	3.85	19	0.001	7.87	19	<0.001	−0.16	19	0.436	No
	Preferred	127 ± 16.00	132 ± 17.50	2.81	20	0.011	5.90	20	<0.001	−0.28	20	0.390	No
	Fast	147 ± 16.00	146 ± 20.00	−0.36	20	0.719	1.49	20	0.077	−2.21	20	0.019	No
AR speed line	Slow	107 ± 16.00	109 ± 18.60	0.95	20	0.354	3.93	20	<0.001	−2.03	20	0.028	Yes
	Preferred	127 ± 16.00	131 ± 19.10	2.56	20	0.019	5.67	20	<0.001	−0.54	20	0.297	No
	Fast	147 ± 16.00	142 ± 22.10	−1.85	20	0.079	−0.19	20	0.576	−3.51	20	0.001	No
AR speed bird	Slow	107 ± 16.00	111 ± 18.40	3.66	20	0.002	7.82	20	<0.001	−0.50	20	0.311	No
	Preferred	127 ± 16.00	132 ± 17.70	2.76	20	0.012	5.45	20	<0.001	0.06	20	0.524	No
	Fast	147 ± 16.00	147 ± 22.70	−0.06	20	0.950	−1.51	20	0.091	−1.51	20	0.073	No
**Step length**													
Real-world stepping targets	Short	52.8 ± 10.24	52.5 ± 9.95	−2.22	17	0.040	28.02	17	<0.001	−32.46	17	<0.001	Yes
	Preferred	68.0 ± 9.99	67.8 ± 10.09	−1.97	18	0.064	27.91	18	<0.001	−31.85	18	<0.001	Yes
	Long	83.4 ± 10.10	83.3 ± 10.10	−1.75	17	0.099	20.69	17	<0.001	−24.18	17	<0.001	Yes
AR dinosaur footprints	Short	52.8 ± 10.20	52.6 ± 10.10	−0.93	17	0.368	16.37	17	<0.001	−18.22	17	<0.001	Yes
	Preferred	68.0 ± 9.99	67.8 ± 10.18	−1.16	18	0.262	16.44	18	<0.001	−18.76	18	<0.001	Yes
	Long	82.8 ± 10.20	82.5 ± 10.30	−1.03	17	0.317	10.90	17	<0.001	−12.96	17	<0.001	Yes
AR dinosaur footprints + sound	Short	53.0 ± 9.99	52.9 ± 9.66	−0.34	18	0.736	19.42	18	<0.001	−20.11	18	<0.001	Yes
	Preferred	68.0 ± 9.99	67.5 ± 10.28	−2.30	18	0.034	14.15	18	<0.001	−18.75	18	<0.001	Yes
	Long	82.9 ± 10.30	82.7 ± 10.60	−1.04	17	0.313	12.84	17	<0.001	−14.92	17	<0.001	Yes

^*^TOST interpretation of equivalence by Lakens (40).

### What is the effect of AR-FOV size on the gait-modifying effect of AR cues?

Differences in AR-FOV size did not influence the gait-modifying effects of cues as there were no main or interaction effects involving the factor Headset on the performed gait adjustments in all cueing tasks [except for the significant Content × Headset interaction for step length, *F*(1.85,25.84) = 8.135, *p* = 0.002, *ƞ*_p_^2^ = 0.368, without any significant post-hoc comparisons].

### What is the effect of AR-FOV size on the head orientation required to interact with AR cues?

In the static trial, the downward head orientation differed between all lines, with larger downward head orientations observed when viewing lines nearby (Figures 2A–C). This was supported by an effect of Distance on head orientation [*F*(2.20,37.40) = 648.662, *p* < 0.001, *ƞ*_p_^2^ = 0.974], with significant differences in head orientation between all lines (*p_bonf_* < 0.001). Post-hoc analyses of the significant Headset × Content × Distance interaction [*F*(6,102) = 2.614, *p* = 0.021, *ƞ*_p_^2^ = 0.133] revealed a trend toward a greater downward head orientation with HL2 compared to ML2 for the first AR line only [*F*(1,17) = 4.248, *p* = 0.055, *ƞ*_p_^2^ = 0.200; Figure 2C], a finding in line with AR-FOV-size differences between headsets. Besides that, there was a main effect of Headset on the required head orientation to interact with step-length cues [*F*(1,17) = 5.285, *p* = 0.034, *ƞ*_p_^2^ = 0.237; Figure 2E], again with a larger downward head orientation for HL2 (41.98 ± 11.34°) than ML2 (32.02 ± 7.45°; *p_bonf_* = 0.034), a finding consistent with the AR-FOV-size differences between headsets.

Finally, the head orientation varied with cueing conditions. For step-length cues, larger downward head orientations with smaller imposed step lengths were observed, as indicated by a main effect of Modulation [*F*(1.92,32.68) = 67.625, *p* < 0.001, *ƞ*_p_^2^ = 0.799], with significant differences in head orientation between levels (all *p_bonf_* < 0.001; Figure 2E). For the speed cues, a larger downward head orientation was found for the slower-than-preferred speed condition only (*p_bonf_* < 0.001), following from a significant main effect of Modulation [*F*(1.72,57.79) = 19.720, *p* < 0.001, *ƞ*_p_^2^ = 0.509]. As expected, there was a profound difference in head orientation between the AR speed-bird condition (−0.36 ± 8.24°) and the AR and real-world speed-cue conditions (34.04 ± 12.37° and 30.17 ± 10.66°, *p_bonf_* < 0.001; Figure 2D). This was supported by a main effect of Content for speed cues [*F*(1.45,27.57) = 132.114, *p* < 0.001, *ƞ*_p_^2^ = 0.874]. The significant Content × Modulation interaction for speed cues [*F*(2.85,54.21) = 10.003, *p* < 0.001, *ƞ*_p_^2^ = 0.345] implied that the factor Modulation only affected head orientation for the two speed-line conditions and not for the AR speed-bird condition (Figure 2D).

## Discussion

The primary objective of this study was to examine if people with PD were able to modify their gait to AR and real-world cue variations and whether such adjustments were equivalent to what was imposed with the cues. Results showed that people with PD can modify their gait speed, step length, and crossing step length to cue variations, for AR and real-world cueing alike. Furthermore, people with PD modulated their step length equivalent to what was imposed by both AR and real-world step-length cues, whereas the performed gait speeds were often slightly different from what was imposed (i.e., participants seemed to walk slightly faster than imposed with slower imposed speeds). This discrepancy in equivalence between step length and gait speed may be caused by less dictating or salient cue information for the speed cues (where participants could vary their distance to the speed line or bird during the trial) compared to the step-length cues that mandated precision stepping throughout the trial and thus constrained variability in task execution. Note, however, that these results applied to both real-world and AR cues. We therefore conclude that people with PD can adjust various aspects of their gait to variations in AR cues, and that they do this as effectively as to real-world cues. These findings, corroborating related work in healthy adults (29, 44), are relevant for recent studies that have already implemented AR cues in training interventions like dual-task training for people with PD, which showed promising results (20, 45).

We further explored the utility of two additional benefits AR cueing may offer, that is (i) by using an AR speed bird in the air to modulate speeds while promoting an upright head orientation and posture and (ii) to augment steps acoustically using action-relevant mud sounds to the AR dinosaur footprints. Regarding the former benefit, we found that participants adjusted their gait speed to the AR speed bird, which did not differ from real-world and AR speed lines. This introduces a new possibility of visual cueing without requiring the individual to look down, which could promote or aggravate a stooped posture in people with PD. A recent study by Retzinger et al. (46) also examined visual AR cues in the air with healthy young adults, in this case for modulating step length through transparent footprints at participant’s chest level accompanied by footprints on the ground. It is, however, unknown whether participants processed spatial information of the footprints in the air, on the ground, or both. The action-relevance, an important factor for effective cueing (47), of such step-length cues in the air is probably much lower [i.e., as the spatial information conveyed by the cues is not directly specifying the actual foot-placement locations (48)] than when participants can directly step onto stepping targets on the ground, for which existing visuolocomotor control mechanisms can be utilized (49–52). The second additional benefit that we explored was adding mud sounds to dinosaur footprints to augment steps. This was anticipated to improve their action relevance. However, this did not further improve their gait-modifying effect compared to dinosaur footprints without acoustic step augmentation. The absence of an additional benefit of the mud sounds may be attributed to an already excellent ability of our participants to position their feet to visual step-length cues, as evidenced by the resulting equivalence between performed and imposed step lengths for all levels of modulation (Figures 3D–F; Table 1).

The secondary objective of this study was to examine differences between the two AR headsets with different AR-FOV sizes in terms of gait-modifying effects and head orientations required to interact with the cues. The modulated gait speeds and step lengths did not differ between the headsets, indicating that the AR-FOV size of state-of-the-art HL2 and ML2 headsets was sufficient for modifying gait with AR cues, thereby overcoming limitations seen in previous studies using first-generation AR headsets with much smaller (vertical) AR-FOV sizes (23, 53). AR-FOV size also had only a small effect on the measured head orientations: a tendency toward a significant difference between headsets was observed for the static task, and only for the nearest AR line (Figures 2B,C), with a larger downward head orientation required for HL2 (with a smaller vertical AR-FOV size of 29°) than for ML2 (55°). Likewise, also a slightly greater downward head orientation was required for HL2 than for ML2 when interacting with step-length cues (Figure 2E). These findings are in line with our previous research on the effect of AR-FOV size (54), contrasting HoloLens 1 (vertical AR-FOV 17.5°) and 2 (29°), where head orientations required for interacting with AR content varied with differences in AR-FOV size, particularly so for content nearby. It is noteworthy, however, that in the current study with state-of-the-art AR headsets with larger vertical AR-FOV sizes, observed head-orientation differences between headsets were much smaller in magnitude and even completely absent between real-world and AR gait-modulating content (except of course for the AR speed-bird condition). Thus, state-of-the-art AR headsets have reached a level where AR-FOV size is no longer a limiting factor for modifying gait with AR cues, nor require greater downward head orientations to get the AR cues into view compared to interacting with similar real-world cues.

We identify some study limitations and implications for future research. First, we were not able to analyze step-height modulation due to task-specific technical issues with the motion-registration system. However, in line with previous research (44), we did observe that people with PD modulated their step height to the different heights of AR and real-world 3D hurdles in the experiment (see also Supplementary Video for a representative participant). Previous research stated that, for some individuals with PD, 3D cues could be more effective than 2D cues for modifying gait and overcoming FoG (16). Even though we could in that regard not provide formal statistical evidence here, we recommend further explorations of the utility of 3D cues for modifying gait and to accommodate heterogeneity in effect of different forms of cueing. Second, our study clearly showed that gait parameters like gait speed and step length could be modulated with AR cues relative to one’s baseline gait pattern. This is encouraging considering earlier research with AR cues showing limited effects on various gait parameters (19, 23–25, 28, 48). AR cues, when delivered through state-of-the-art AR headsets which have a sufficiently large vertical AR-FOV size, may thus be used to improve Parkinsonian gait. For example, step-length-modulating AR cues may assist in (i) increasing the typically short step lengths seen in people with PD (55) and (ii) alleviating FoG, considered to be one of the most disabling symptoms in people with PD, elevating fall risk and reducing quality of life (56). In doing so, one could ultimately take advantage of the flexibility (selecting the most effective type of cue) and personalization (tailoring the cues to individual’s gait characteristics) potential of AR cueing, as cueing is not a one-size-fits-all principle (1, 17). Additional benefits that AR-cueing applications may offer besides flexibility and personalization are (i) multimodality (e.g., visual cues, auditory cues, or both), (ii) cue activation [e.g., making use of headset-data features (20), on-demand activation with voice commands (23) or intelligent open-loop vs. closed-loop cueing (57)] and (iii) spatial awareness (e.g., merging visual cues to features in mapped environments). The latter seems particularly useful when transitioning from the lab (as in the current study) toward implementation in the home environment of people with PD, as was already explored by Geerse et al. (23). These future studies should also consider user experience and feedback (e.g., usability, comfort, adverse events) of a diverse range of individuals with PD for the long-term use of AR cueing applications in real-world environments.

To conclude, this study revealed that people with PD can adjust various aspects of their gait to variations in AR cues as effectively as to real-world cues and that the AR-FOV size of state-of-the-art AR headsets is sufficiently large for modifying gait without affecting the head orientations required to interact with AR cues.

## Data availability statement

The original contributions presented in the study are included in the article/Supplementary material, further inquiries can be directed to the corresponding authors.

## Ethics statement

The studies involving humans were approved by the Accredited Medical Research Ethics Committees United (MEC-U), the Netherlands (R22.076, NL82441.100.22). The studies were conducted in accordance with the local legislation and institutional requirements. Participants provided written informed consent for participation in this study. Written informed consent was obtained from the individual(s) for the publication of any potentially identifiable images or data included in this article.

## Author contributions

EH: Data curation, Formal analysis, Investigation, Methodology, Visualization, Writing – original draft, Writing – review & editing. DG: Writing – review & editing, Conceptualization, Supervision. AD: Data curation, Writing – review & editing. JS: Formal analysis, Writing – review & editing. MR: Conceptualization, Funding acquisition, Supervision, Writing – review & editing.
