# Supplementary material for: Gait-modifying effects of augmented-reality cueing in people with Parkinson’s disease
Source: Front Neurol. 2024 Apr 9;15:1379243. doi: 10.3389/fneur.2024.1379243 (PMC11037397; doi:10.3389/fneur.2024.1379243)
Supplement: Supplementary file 1 [file Table_1.DOCX]

**Supplementary Table 1:** Participant characteristics

| Characteristics | HoloLens 2 (n=11) | Magic Leap 2 (n=10) | Statistic |
| --- | --- | --- | --- |
| Age (years) | 63 ± 8.6 [51-74] | 69 ± 8.3 [53-82] | *t*(19)=-1.759*, p*=0.095 |
| Sex, M/F | 7/4 | 8/2 | *X^2^*(1)=0.687, *p*=0.407 |
| Time since diagnosis (years) | 6.3 ± 5.7 [1-20] | 8.6 ± 3.8 [4-15] | *U*=34.500, *p*=0.157 |
| Modified Hoehn & Yahr stage, 2/2.5 | 7/4 | 6/4 | *X^2^*(1)=0.029, *p*=0.864 |
| MoCA score | 26.2 ± 3.4 [18-29] | 28.1 ± 2.2 [23-30] | *U*=30.000, *p*=0.080 |
| LEDD (max. mg/day) | 844.9 ± 673.8 [125-2400] | 939.9 ± 488.2 [375-1738] | *t*(19)=-0.367*, p*=0.718 |
| FoG, yes/no* | 4/7 | 7/3 | *X^2^*(1)=2.376, *p*=0.123 |
| MDS-UPDRS part III score | 30.5 ± 13.8 [13-61] | 31.6 ± 8.2 [21-46] | *t*(19)=-0.210*, p*=0.836 |
| Fall history (number of falls in the past year) | 3.1 ± 3.5 [0-10] | 2.2 ± 3.4 [0-10] | *U*=66.500, *p*=0.424 |

Data are mean ± SD [range] unless indicated otherwise. M = male, F = female, MoCA = Montreal Cognitive Assessment, LEDD = Levodopa Equivalent Daily Dose, FoG = Freezing of gait, MDS-UPDRS = MDS-Unified Parkinson’s disease rating scale. *The presence of FoG is defined by a non-zero score on the New Freezing of Gait Questionnaire.
